# Sperm kinematic subpopulations of the American crocodile (*Crocodylus acutus*)

**DOI:** 10.1371/journal.pone.0248270

**Published:** 2021-03-09

**Authors:** Anthony Valverde, Olivier Castro-Morales, Mónica Madrigal-Valverde, Marlen Camacho, Vinicio Barquero, Carles Soler, Eduardo R. S. Roldan

**Affiliations:** 1 Costa Rica Institute of Technology, School of Agronomy, Cartago, Alajuela, Costa Rica; 2 Department of Cellular Biology, Functional Biology and Physical Anthropology, University of Valencia, Burjassot, Spain; 3 Department of Biodiversity and Evolutionary Biology, Museo Nacional de Ciencias Naturales (CSIC), Madrid, Spain; University of Hyderabad, INDIA

## Abstract

There has been very limited use of computer assisted semen analysis (CASA) to evaluate reptile sperm. The aim of this study was to examine sperm kinematic variables in American crocodile (*Crocodylus acutus*) semen samples and to assess whether sperm subpopulations could be characterized. Eight ejaculates (two ejaculates/male) from four sexually mature captive crocodiles were obtained. An ISAS®v1 CASA-Mot system, with an image acquisition rate of 50 Hz, and ISAS®D4C20 counting chambers were used for sperm analyses. The percentages of motile and progressively motile spermatozoa did not differ among animals (P > 0.05) but there was a significant animal effect with regards to kinematic variables (P < 0.05). Principal component (PC) analysis revealed that kinematic variables grouped into three components: PC1, related to velocity; PC2 to progressiveness and PC3 to oscillation. Subpopulation structure analysis identified four groups (P < 0.05), which represented, on average, 9.8%, 32.1%, 26.8%, and 31.3% of the total sperm population. Males differed in the proportion of sperm in each of the kinematic subpopulations. This new approach for the analysis of reptile sperm kinematic subpopulations, reflecting quantifiable parameters generated by CASA system technology, opens up possibilities for future assessments of crocodile sperm and will be useful in the future development of assisted reproduction for these species.

## Introduction

The American crocodile [*Crocodylus acutus* (Cuvier, 1807)] is a large, oviparous and aquatic (fresh, brackish, or salt water) reptile distributed in subtropical and tropical zones [1]. *C*. *acutus* has a wide distribution that extends from the southern tip of Florida, United States, and the Caribbean islands to the Yucatan Peninsula in Mexico, then southward to Colombia and Venezuela, and on the Pacific from northern Sinaloa, Mexico, to northern Peru [2]. In Costa Rica, *C*. *acutus* is mainly distributed on both coastlines in large rivers and streams, often in brackish water near the mouths of rivers, as well as in salt and freshwater marshes, swamp forests and mangrove swamps, at elevations below 200 m [3,4]. Although typically associated with brackish estuaries and large rivers and streams, C. *acutus* occasionally ventures into marine environments [4], indicating some tolerance of saltwater [5,6]. *C*. *acutus* is found in Appendix II of CITES [7], in the vulnerable category, due to overexploitation and habitat loss [8]. In some countries, such as Costa Rica, Cuba, Mexico and Venezuela, protection has resulted in substantial recovery, but overall numbers are still depleted in Colombia and Ecuador [9].

The reproductive biology of crocodiles has recently attracted attention in wildlife farming and agro-ecoturism activities. Semen collection has been performed post-mortem in the crocodile [10]. Interest in the development of assisted breeding technology of captive animals [11] has led to a refinement of semen collection protocols and characterization of sperm variables [12–15]. A common method of semen collection in wildlife is electroejaculation but this approach is highly stressful for crocodiles and induces urine contamination [13]. A new technique consisting of digital manipulation in sedated crocodiles has been introduced and this would allow studies following animal welfare protocols [16].

To the best of our knowledge, sperm kinematic evaluation in the *C*. *acutus*, with the help of computer assisted semen analysis (CASA) systems, has never been reported. Assessment of sperm motility in different species, at reproductive research centres, is now commonly performed with computer assisted semen analysis (CASA-Mot) technologies which allows for objective and accurate assessment of sperm kinematics [17–20]. The CASA systems provide information based on values of thousands of sperm tracks in a sample [21]. It has been described that ejaculates are heterogenous with regards to patterns of sperm motility and, using CASA-Mot systems, subpopulations of spermatozoa that exhibit different kinematic patterns have been identified. The biological significance of these different sperm subpopulations is still being studied in different species with a view to assessing their fertilizing capacity [22].

Studies of sperm subpopulations have been conducted mainly on semen from mammalian species [23,24], namely cattle [25–27], sheep [28,29], pigs [30,31], stallion [32], ram [29,33], fox [34], cat [35], and primates [36], with CASA systems showing that they are a most reliable and accurate method for studying sperm subpopulation distribution. Characterization of sperm subpopulations has also received attention in fishes [37–39]. A previous study in the brown caiman (*Caiman crocodilus fuscus*) has revealed that kinematic sperm subpopulations can also be identified in this species [15].

This report is aimed at establishing conditions for the collection and analyses of *C*. *acutus* spermatozoa, with particular interest in the characterization of sperm swimming patterns and kinematic subpopulation structure.

## Materials and methods

### Study site

The present study was conducted at the crocodile management and exhibition facilities associated to the Scientific Ecotourism Project (EcoTEC) based at the School of Agronomy, Costa Rica Institute of Technology, San Carlos Campus, Alajuela, Costa Rica (10°21’52” N, 84°30’31’ W). The facility is located at an altitude of 170 m above sea level, in a tropical wet forest with a basal altitudinal floor, according to the Holdridge life zones system [40]. According to data recorded at the closest weather station (069567, St Clara, University Campus), the crocodile facility has annual minimum and maximum temperatures of 21.7°C and 30.7°C, respectively and a relative humidity of 88.5%, with a rainfall rate of 3321.1 mm per year.

### Animals

This study was conducted under conditions that comply with laws and regulations controlling experiments on live animals in Costa Rica and did not require approval by the animal research committee of the Costa Rica Institute of Technology. This study was conducted with the approval of the National System of Conservation Areas (SINAC-Costa Rica) and Arenal Huetar Norte Conservation Area (ACAHN) Scientific Purposes Permit SINAC-ACAHN-SCH-759-18.

Four sexually mature healthy male crocodiles were used as semen donors in this study. The animals were housed together with nine females in the same pond. All the crocodiles were estimated to be over 25 years old and they were sourced from the EcoTEC project. The animals were incorporated to the project due to relocation and rescue since 2002. The process with animals for habitat release was authorized for National System of Conservation Areas. All the animals were identified by microchip or scales slitting, for their biological monitoring. More details on crocodile identification are presented in Table 1. The crocodiles were fed with pieces of lean meat (pork, chicken, beef—which can be fed on the bone in larger adults), that was supplemented with additional calcium at 2.0 to 2.4% dry matter.

**Table 1:** American crocodile (*Crocodylus acutus*) semen donor information.

Male	Croc ID	Age (years)*	HTL (cm)	SV (mL)	SD
1	C076	25+	390	1.0	0.28
2	C286	25+	335	1.5	0.21
3	C129	25+	323	1.5	0.07
4	C800	25+	315	1.0	0.07

Animals were housed in the same pond and all males were included in the EcoTEC project.

*Unknown age when wild caught (25+); HTL: Head to tail length; SV: Mean semen volume; SD: Standard deviation of semen volume.

### Crocodile restraining

Semen was collected from restrained crocodiles, without sedation. Animal handling was carried out as described previously [41], with some modifications as per criteria and skills of the local team. Materials used for capture ensured that restrain was secure at all times and that it did not softened when used in water or, subsequently, when animals were taken to the place of semen collection. Front and hind legs were carefully tied caudal to the shoulders and pelvis of the animal to prevent injury to semen collector or the crocodile. All animal restraint procedures were carried out without any incident.

### Semen collection

**Fig 1:**
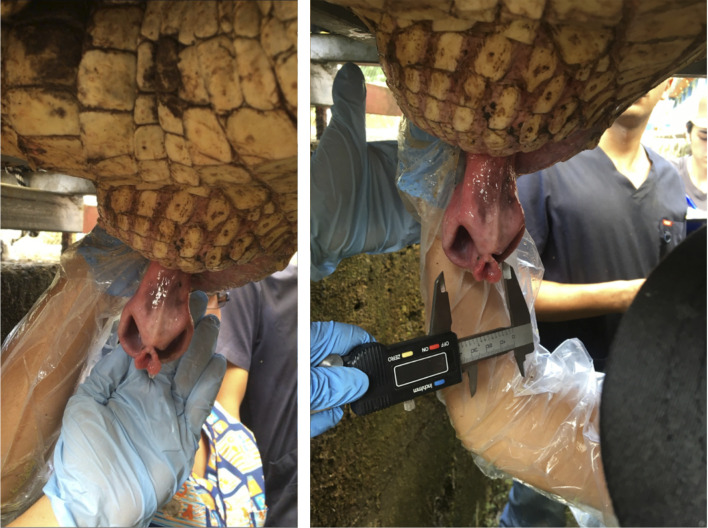
American crocodile (*Crocodylus acutus*) sperm collection by manipulation and digital massage of the penis and ductus deferens, introducing a gloved hand in the opening cloaca.

Samples were obtained during the period December 2015—May 2016 when the animals show courtship and mating behaviour (usually from September to January, during the rainy season). Ejaculate collection was carried out by digital manipulation as previously described [13] (Fig 1). A gloved hand was introduced into the cloaca, with a caudal direction, to gently exteriorize the phallus; once the phallus was exteriorized the fore and index fingers were used to gently massage-stroke the terminal portions of the ductus deferens immediately cranial to the urodeum. Gloves were latex-free and did not contain any spermicide. Semen collection was performed twice in each animal and there was a gap of two weeks between each collection. Following massage, semen flowed down the sulcus of the phallus and was carefully lavaged into a collection vessel. Small volumes of ejaculate (e.g., 1.0 mL) were recovered into a 1.5 mL Eppendorf^®^ microtube (Sigma-Aldrich, St. Louis, MO, USA) aided by use of a micropipette fitted with a 10–100 μL pipette tip. Semen was recovered from the sulcus with approximately 100 μL of buffered Dulbecco’s phosphate-buffered saline (DPBS, pH 6.8, Sigma-Aldrich).

### Assessment of sperm variables

The pH of undiluted semen was determined in eight ejaculates using narrow range pH paper strips (± 0.3 pH units; Sigma-Aldrich). A further dilution (1:10 in DPBS diluent) was used for motility and kinematic assessments. For the analysis of motility and kinematic variables, ISAS^®^D4C20 disposable counting chambers (Proiser R+D, S.L., Paterna, Spain) were used after being pre-warmed to 25°C [15]. After thorough mixing of the diluted semen samples, 3 μL of diluted semen were placed in the counting chamber tracks by capillarity. Analyses were conducted with the CASA-Mot system ISAS^®^v1 (Integrated Semen Analysis System, Proiser R+D, Paterna, Spain) fitted with a video-camera (Proiser 782M, Proiser R+D), with a frame rate of 50 frames per second (fps) and a final resolution of 768 x 576 pixels. The camera was attached to a microscope UB203 (UOP/Proiser R+D) with a 1x eyepiece and a 10X negative-phase contrast objective (AN 0.25) and an integrated heated stage maintained at 25 ± 0.5°C. Sperm concentration (x10^9^ / mL) was estimated in the CASA-Mot system after accounting for the initial dilution of the semen sample.

### Kinematic analysis

CASA analyses were performed in seven microscope fields on a total of at least 600 cells per sample. The CASA-Mot variables assessed in this study included: straight-line velocity (VSL, μm / s), corresponding to the straight line from the beginning to the end of the track; curvilinear velocity (VCL, μm / s), measured over the actual point-to-point track followed by the cell; average path velocity (VAP, μm / s), the average velocity over the smoothed cell path; amplitude of lateral head displacement (ALH, μm), defined as the maximum of the measured width of the head oscillation as the sperm swims; beat-cross frequency (BCF, Hz), defined as the frequency with which the actual track crosses the smoothed track in either direction; motility (%), the percentages of motile- and progressively motile spermatozoa, corresponding to spermatozoa swimming rapidly forward in a straight line (assessed as straightness index ≥45%; VAP ≥25 μm / s). Three progression ratios, expressed as percentages, were calculated from the velocity measurements described above: linearity of forward progression [LIN = (VSL/VCL) x100], straightness [STR = (VSL/VAP) x100], and wobble [WOB = (VAP/VCL) x100].

### Statistical analysis

The data obtained from the analysis of all sperm parameters were first assessed for normality and homoscedasticity by using Shapiro-Wilks and Levene tests. A normal probability plot was used to assess normal distribution. Multivariate procedures were performed to identify sperm subpopulations from the set of sperm motility data. All the values for kinematic variables were standardized to avoid any scale effect. The first process was to perform a principal component analysis (PCA) of these data to derive a small number of linear combinations (PCs) that still retained as much information as possible from the original variables. The number of principal components (PC) used in the next process of the analysis was determined from the Kaiser criterion, namely selecting only those with an eigenvalue (variance extracted of each PC) > 1. Furthermore, Bartlett’s sphericity test and the KMO (Kaiser-Meyer-Olkin) test were performed [42]. As a rotation method, the varimax method with Kaiser normalization was used [43]. The second process was conducted to perform a non-hierarchical analysis with the k-means model that uses Euclidean distances from the quantitative variables after standardization of these data, so the cluster centres were the means of the observations assigned to each cluster [44]. The multivariate k-means cluster analysis was made to classify the spermatozoa into a reduced number of subpopulations (clusters) according to their kinematic variables. In the final process, to determine the optimal number of clusters, the final centroids were clustered hierarchically using the Ward method [45]. Thus, the clustering procedure enables for the identification of sperm subpopulations because each cluster contributed to a final cluster formed by the spermatozoa linked to the centroids. An ANOVA was applied to evaluate statistical differences in the distributions of observations (individual spermatozoa) within subpopulations and then a generalized linear model (GLM) procedure was used to determine the effects on the mean kinematic variable values defining the different sperm subpopulations (i.e., the cluster centres). Differences between means were analyzed by a Bonferroni test. Results are presented as mean ± standard error of the mean (SEM). Statistical significance was considered at P < 0.05. All data were analyzed using IBM SPSS package, version 23.0 for Windows (SPSS Inc., Chicago, IL, USA).

## Results

The total time of handling of each animal for semen collection typically did not exceed 30 minutes. All semen collection procedures were conducted without any incident. No attempt to collect semen failed or proved particularly difficult. Collections took place during the period when animals exhibited courtship behaviours. From observations of crocodile behaviour at the EcoTEC project facilities since 2008, it was concluded that *Crocodylus fuscus* express courtship and mating behaviours from September to January (rainy season), the females deposit eggs and incubate the eggs from January to April (dry season) and subsequently they exhibit births from May to July, during the rainy season (J. Bolaños, personal communication).

### Descriptive analysis

The mean (± SEM) sperm concentration (x10^9^ / mL) of the samples was 2.26 ± 0.21 with a range of 0.29–3.60. The mean of total number of sperm (x10^9^ spermatozoa) per male was 2.9 (male 1); 0.4 (male 2); 3.6 (male 3) and 3.2 (male 4). Mean (± SEM) pH of the samples was 6.8 ± 0.1.

### Sperm motility and kinematics

The kinematic variables corresponding to the whole sperm population are described in Table 2. There was no animal effect (P > 0.05) on the percentages of motile and progressively motile spermatozoa. The kinematic variables that indicated greatest variability were VSL, LIN and BCF, with coefficients of variation of 82.5%, 69.2% and 84.0%, respectively. There were differences (P < 0.05) in values among animals for kinematics variables, with VCL, WOB and ALH being the most variable (Table 3).

**Table 2:** Sperm kinematics variables (mean and dispersion) in eight ejaculates (two ejaculates/male) of four American crocodiles (*Crocodylus acutus*).

Parameter	Mean ± SEM	SD	Min	Max	Q1	Q3
TMOT	30.04±0.63	5.72	19.00	41.00	26.00	34.00
PMOT	14.21±0.35	3.16	6.00	22.00	12.00	16.00
VCL	43.90±0.22	20.66	6.10	105.00	28.10	56.60
VSL	13.67±0.12	11.26	0.90	95.20	6.70	17.10
VAP	21.2±0.1	10.5	3.0	97.7	14.20	25.70
LIN	35.4±0.3	24.5	1.0	100.0	16.20	50.60
STR	61.34±0.3	28.2	1.8	100.0	40.30	84.60
WOB	53.0±0.2	20.2	8.0	100.0	37.70	66.40
ALH	2.4±0.01	1.0	0.0	6.5	1.70	3.00
BCF	2.5±0.02	2.1	0.0	14.0	1.00	4.00

Number of motile cells = 8 640. TMOT = percentage motile spermatozoa; PMOT = percentage progressively motile spermatozoa; VCL = curvilinear velocity (μm / s); VSL = straight-line velocity (μm / s); VAP = average path velocity (μm / s); LIN = linearity of forward progression (%); STR = straightness (%); WOB = wobble (%); ALH = amplitude of lateral head displacement (μm); BCF = beat-cross frequency (Hz); SEM = standard error of the mean. SD: Standard deviation; Min = minimum value; Max = maximum value. Q1 = lower quartile. Q3 = upper quartile.

**Table 3:** Motility and sperm kinematic variables (mean ± SEM) in American crocodiles (*Crocodylus acutus*) (two ejaculates/male).

	Male			
Parameter	1	2	3	4
TMOT	31.05±1.33	29.14±1.26	29.70±1.29	30.32±1.23
PMOT	15.26±0.72	13.43±0.69	13.95±0.70	14.27±0.67
VCL	31.55±0.48^a^	38.09±0.19^b^	43.05±0.27^c^	57.41±0.40^d^
VSL	12.11±0.19^a^	13.03±0.22^ab^	13.18±0.23^b^	15.57±027^c^
VAP	17.21±0.19^a^	19.99±0.17^b^	20.69±0.19^b^	25.44±0.24^c^
LIN	44.17±0.52^a^	35.23±0.56^b^	32.54±0.54^c^	30.88±0.46^c^
STR	68.07±0.54^a^	60.71±0.67^b^	59.74±0.66^bc^	57.69±0.55^c^
WOB	61.20±0.43^a^	53.39±043^b^	50.22±0.43^c^	48.24±0.39^d^
ALH	1.92±0.02^a^	2.22±0.01^b^	2.42±0.01^c^	2.99±0.02^d^
BCF	1.85±0.04^a^	2.53±0.05^b^	2.70±0.05^b^	2.99±0.04^c^

TMOT = percentage motile spermatozoa; PMOT = percentage progressively motile spermatozoa; VCL = curvilinear velocity (μm / s); VSL = straight-line velocity (μm / s); VAP = average path velocity (μm / s); LIN = linearity of forward progression (%); STR = straightness (%); WOB = wobble (%); ALH = amplitude of lateral head displacement (μm); BCF = beat-cross frequency (Hz); SEM = standard error of the mean. Number of cells = 8 640. Total number of cells for each male: Male 1 = 2153, male 2 = 1089, male 3 = 1819, male 4 = 2859. ^a-d^ Different superscripts within a row indicate differences among animals P <0.05.

### Principal components analysis and subpopulation structure

Results from principal component analysis indicated there were three PCs: Velocity (PC1) represented by VCL, VAP, ALH and BCF, with a greater effect of VCL (eigenvector of 0.891). PC2 represented by VSL, LIN, and STR, is referred to as progressiveness, with a greater effect of STR (eigenvector of 0.918). Finally, PC3, represented by WOB, LIN and VAP, termed oscillation, was mainly related to WOB (eigenvector of 0.954), with a total variance of 85.08% explained. These results indicated that sperm velocity has a relatively greater effect on the total variance than the other variables (Table 4).

**Table 4:** Eigenvectors of principal components (PCs) for American crocodile (*Crocodylus acutus*) sperm kinematic parameters.

Principal component*	PC1	PC2	PC3
VCL	0.891		
VSL		0.859	
VAP	0.847		0.428
LIN		0.807	0.474
STR		0.918	
WOB			0.954
ALH	0.861		
BCF	0.511		
Variance explained (%)	35.44	29.70	19.94

Total variance explained = 85.08%.

*Expresses the more important variables in each PC. Only eigenvectors > 0.4 are presented. VCL: Curvilinear velocity; VSL: Straight-line velocity; VAP: Average path velocity; LIN: Linearity of forward progression; STR: Straightness; WOB: Wobble; ALH: Amplitude of lateral head displacement; BCF: Beat-cross frequency.

**Fig 2:**
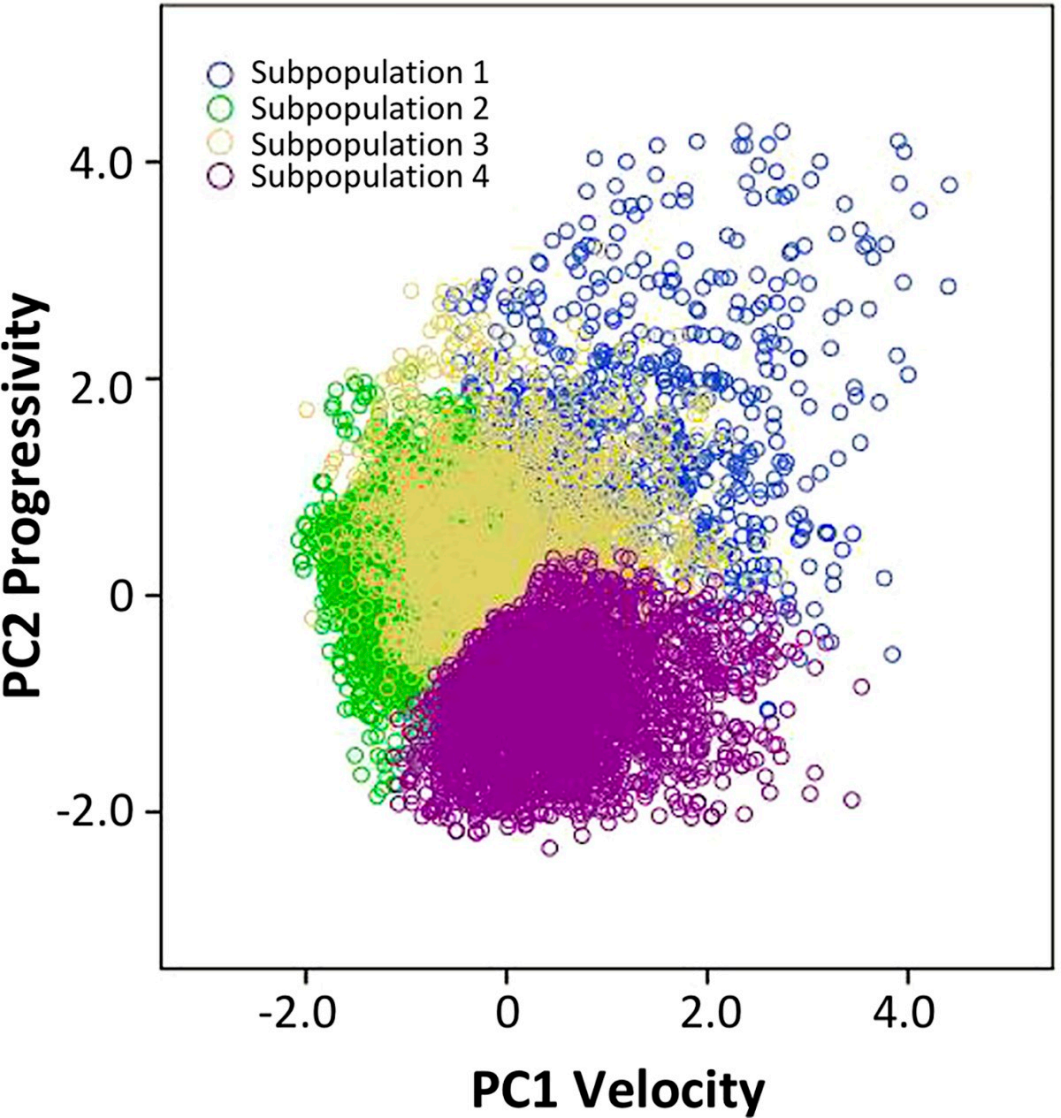
Distribution of sperm subpopulations of American crocodile (*Crocodylus acutus*) according to their principal components values.

**Fig 3:**
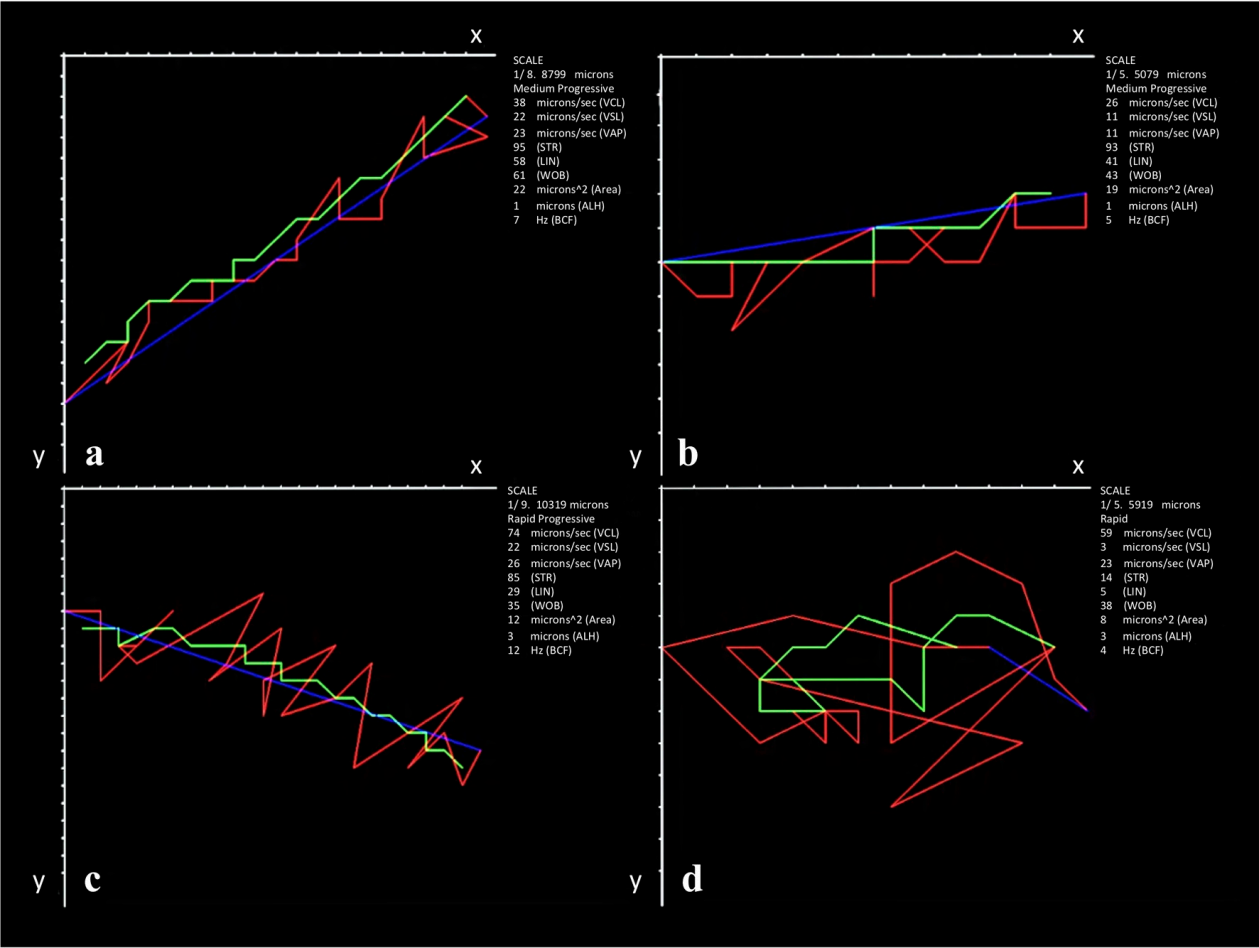
Representative trajectories by subpopulation of American crocodile (*Crocodylus acutus*) sperm analyzed with ISAS®v1 CASA-Mot system. a: Rapid progressive (SP1); b: Slow progressive (SP3); c: Medium non-progressive (SP4); d: Slow non-progressive (SP2). Lines: Blue = VSL; Red = VCL; Green = VAP. VCL = curvilinear velocity (μm / s); VSL = straight-line velocity (μm / s); VAP = average path velocity (μm / s). SP: Subpopulation.

The data from cluster analysis revealed four subpopulations (SPs) (Fig 2). The kinematic values corresponding to each subpopulation were characterized as: (a) rapid progressive (SP1), which showed the highest VSL (37.14±0.55 μm / s), LIN and STR (65.21±0.81; 83.31±0.57 μm / s respectively), comprised 9.8% of the total cells; (b) slow progressive (SP2), exhibiting low velocity with high progressivity cells (LIN = 51.89±0.37%; STR = 74.06±0.33%); (c) medium non-progressive (SP3), which included cells with moderate velocity and progressivity (LIN = 29.91±0.34%), being 26.8% of the total cells, and (d) slow non-progressive (SP4), which was characterized by the lowest velocity and progressivity (VSL = 7.04±0.09 μm / s; LIN = 13.98±0.18%). The subpopulation SP2 showed the highest proportion of cells (32.1%), followed by SP4 with 31.3% of total sperm cells (Fig 3, Table 5).

**Table 5:** Sperm subpopulations (SP) for kinematic variables (means ± SEM) in American crocodile (*Crocodylus acutus*).

	Subpopulation and number of cells (%)			
Kinematic descriptor	SP1	SP2	SP3	SP4
	847 (9.8)	2775 (32.1)	2316 (26.8)	2702 (31.3)
VCL	58.94±0.67^a^	23.74±0.14^b^	53.08±0.37^c^	52.02±0.33^c^
VSL	37.14±0.55^a^	11.92±0.10^b^	14.93±0.16^c^	7.04±0.09^d^
VAP	40.84±0.44^a^	15.60±0.09^b^	17.72±0.13^c^	23.93±0.16^d^
LIN	65.21±0.81^a^	51.89±0.37^b^	29.91±0.34^c^	13.98±0.18^d^
STR	83.31±0.57^a^	74.06±0.33^b^	75.53±0.42^c^	29.22±0.32^d^
WOB	71.43±0.61^a^	67.58±0.30^b^	34.52±0.20^c^	48.13±0.29^d^
ALH	2.94±0.03^a^	1.62±0.01^b^	2.69±0.02^c^	2.92±0.02^a^
BCF	3.21±0.09^a^	1.52±0.03^b^	2.39±0.04^c^	3.54±0.04^d^

VCL = curvilinear velocity (μm / s); VSL = straight line velocity (μm / s); VAP = average path velocity (μm / s); LIN = linearity of forward progression (%); STR = straightness (%); WOB = wobble (%); ALH = amplitude of lateral head displacement (μm); BCF = beat-cross frequency (Hz); SEM = standard error of the mean.

^a-d^ Different superscripts within row indicate significant differences among subpopulations. P <0.05.

Table 6 shows that proportions of sperm cells in each subpopulation were different between males and between subpopulations in each male. The sperm subpopulations are unevenly distributed for each male. The sperm subpopulations with highest number of cells were associated with a specific male, [male 1: SP2 (48.44%); male 2: SP3 (33.17%); male 3: SP4 (41.84%); male 4: SP1 (38.27%)]. However, subpopulations with lower percentages of cells in both male 1 and 3 were associated to SP1 (Table 6). Odds-ratio analysis indicates that the distribution of frequencies or spermatozoa of the sperm subpopulations is not the same for all the males (Table 7). The comparison between SP1 and SP2 (odds-ratio value = 6.68) indicates that the ratio between the frequencies of SP2 and SP1 is 6.68 times higher in male 1 than in male 2. This implies that in terms of probability (likelihood to occur), there is an 80–90% likelihood of finding sperm cells of SP2 in male 1 in comparison to male 2 (Table 7). Comparison between SP1 and SP3 showed an odds-ratio value of 4.99, whereas the ratio in which SP3 appears in male 2 is 5.99 times greater than in the male 4. Finally, the ratio in which SP4 appears in male 3 is 4.37 times greater than in the male 4 (Table 7).

**Table 6:** Percentage of sperm cells in each kinematic subpopulation (SP) characterized in semen from four American crocodile (*Crocodylus acutus*) males.

	% sperm in each subpopulation			
Male	SP1	SP2	SP3	SP4
1	9.06 ^a^^α^	48.44 ^b^^α^	25.41 ^c^^α^	17.09 ^d^^α^
2	26.42 ^a^^β^	21.17 ^b^^β^	33.17 ^c^^β^	19.24 ^b^^β^
3	14.73 ^a^^γ^	21.39 ^b^^β^	22.05 ^b^^γ^	41.84 ^c^^γ^
4	38.27 ^a^^ε^	27.25 ^b^^ε^	9.62 ^c^^ε^	24.87 ^d^^ε^

Each row indicates the percentage of spermatozoa in each sperm subpopulation. For characterization of each subpopulation see text, Table 5 and Fig 3. Total number of cells for each male: Male 1 = 2153, male 2 = 1089, male 3 = 1819, male 4 = 2859.

^a, b, c, d^ Superscript indicates differences within row regarding sperm subpopulation.

^α, β, γ, ε^ Superscript indicates differences within column for each animal, chi squared (χ^2^) test, P < 0.05.

**Table 7:** Odds-ratio values of kinematic subpopulations based a pair-wise comparison among four American crocodile (*Crocodylus acutus*) males*.

	Comparison between subpopulations					
Male compared	1–2	1–3	1–4	2–3	2–4	3–4
1–2	6.68	2.23	2.59	0.33	0.39	1.16^ns^
1–3	3.68	1.87	0.66	0.51	0.18	0.35
1–4	7.51	11.16	2.90	1.49	0.39	0.26
2–3	0.55	0.84^ns^	0.26	1.52	0.46	0.31
2–4	1.13^ns^	4.99	1.12^ns^	4.44	1.00^ns^	0.22
3–4	2.04	5.95	4.37	2.92	2.14	0.73

ns: Not significant, chi squared (χ^2^) test

* P <0.05.

## Discussion

To the best of our knowledge, the results of this work represent the first study on sperm kinematic parameters of *C*. *acutus* semen, examined with a computer assisted semen analysis (CASA) system. Semen analysis is important to assess the reproductive potential of males, and CASA technology provides an objective and repeatable assessment of the number of motile sperm cells in a sample, as well as for measuring several kinematic variables [21,46]. Previous studies indicated that the percentage of motile spermatozoa and their swimming descriptors are directly correlated with fertilization success [47,48]. This can allow identification of samples with poor sperm motility and can also be a useful technique in predicting the most desirable males for artificial insemination programmes based on sperm motility and kinematic variables. *C*. *acutus* is a vulnerable species and the present study, although limited because of the number of males available for study, represents an initial contribution to a better characterization of semen from this species.

The percentage of motile spermatozoa in ejaculates of *C*. *acutus* varied from 19 to 41%. The mean (± SD) percentage of motile spermatozoa reported in this study (30.0 ± 5.7%) was lower than results found in a related species (*Crocodylus porosus*: 45.0 ± 17.56% [12]; 50.7 ± 4.2% [13]; 63.4 ± 3.2% [16]). In others species, higher values were recorded in alligators, *Alligator mississippiensis* (75–85%) [49,50], and *Caiman crocodilus fuscus* (45.9%) [15]. Values of 80–90% were noted in spectacled caimans (*Caiman crocodilus* [51]) and 57.3% in the leopard tortoise [52]. The percentage progressively motile spermatozoa in *C*. *acutus* varied from 6.0 to 22%, with a mean value of 14.2 ± 3.2%. The percentage progressively motile spermatozoa, which was defined as to spermatozoa swimming forward quickly in a straight line (STR ≥45%; VAP ≥25 μm / s), was not reported in other species.

In the past, the whole population of spermatozoa in an ejaculate has been considered as a normal distribution model and the parameters corresponding to motility (or other sperm variables) represented by some measure of dispersion around a central value, usually mean and standard deviation. However, in recent years, by considering the quantitative data obtained from CASA systems, different authors have proposed that the actual distribution of sperm cell characteristics is not uniform, not normally distributed, but structured in well-defined subpopulations [26,34,37,53–57]. In the present study, we have analyzed the sperm kinematic variables of different crocodile ejaculates. Based on cluster analysis, sperm were classified into four clusters, with the following characteristics: One cluster included sperm cells with high velocity and highly linear cells (high VSL, LIN and STR) and was considered SP1 or “rapid progressive”. The second showed low VCL, VSL, VAP and high linearity, and was considered as SP2 or “slow progressive”. The third showed moderate velocities (VCL, VSL and VAP) but low linearity and was defined as SP3 or “medium non-progressive”. Finally, the last one was related to the lowest velocities (VSL, and VAP) and lowest linearity (LIN, STR) and was designated as SP4 or “slow non-progressive” subpopulation.

Regarding the sperm subpopulation distribution in relation to kinematics variables, the question that arises is whether there is a pattern of distinct sperm subpopulations. It seems likely that the patterns of cell cluster formation are determined by sperm velocity, progressive movement and sperm oscillation pattern [15,24,31,34,54,58,59]. In pig [31], fox [34] and caiman [15], sperm subpopulations with a common pattern concerning movement and progressivity of spermatozoa have been identified. The progressiveness of spermatozoa was associated with their velocity, whereas non-progressive movement associated with lower velocity. This raises the question as to what is the role of non-progressive cell subpopulations of reptilian sperm and their relationship with subpopulations of progressive cells. Variation in subpopulation structure among males could be explained by differences in spermatogenesis or post-testicular modifications of spermatozoa, which are believed to be under genetic control [47] and are known to be affected by a series of factors. An additional possibility is that differences between subpopulations may relate to variation in an individual’s strategy and that a strict genetic basis for this trait must be viewed with caution. More work is needed to understand the meaning of these results, but it could be hypothesized that a combination of genetic and external factors may be responsible for the distribution of spermatozoa in different subpopulations of an individual [60–64]. Although there are differences between animals, important questions still arise with regards to whether the proportions of spermatozoa in each subpopulation remain constant over time or if they vary with time in a given male. This could indicate how plastic the character may be and if males can manipulate it in some way, or could respond to different stimuli by varying the proportion of cells in each subpopulation. The methods used to characterize sperm subpopulations can discriminate different cell patterns in males [15,37,56,58,65,66], so it is more likely that some subpopulations are more represented in some animals than others. This could explain part of the individual variation shown by each male and reinforce the idea of individual sperm selectivity and competence, but it is still unclear whether a strategy is maintained over time or what its adaptive value could be.

It is possible that semen collection techniques used here do no equate to semen release and transfer occurring during natural mating because, among other things, there is some degree of stress due to animal restraint. Nevertheless, the use of biomimetic technologies employed in semen collection could be an option to mimic a naturally occurring event [67]. For example, in dogs, manually-collected semen, used either raw or chilled, is of equivalent quality to that resulting from natural mating [68]. When electrejaculation is performed to collect semen from iguanas, semen characteristics are similar to semen samples obtained from other reptiles [69]. It is not currently known how much the manual collection method used here in crocodiles equates to semen transfer during natural copulation, or whether semen differs between captive animals or those in natural populations, and this should be explored in the future. In any case, crocodilian research in the framework of conservation projects in captivity, involve traditional methods of capture and restraint and it may be difficult to overcome this limitation. Furthermore, the use of drugs for animal handling and semen collection may cause additional stress and risks to animal health. In our work, we employed restraint methods for immobilization of non-sedated male crocodiles and successfully managed to collect semen by tactile manipulation minimizing captive management and stress in the males. In the future, it may be possible to employ in crocodilians some technologies currently used in livestock, such as endoscopy and ultrasound procedures to examine the location of the oviduct for future artificial insemination [70] and enable to track the follicular development and study the season to determine the best time for insemination.

*C*. *acutus* is currently listed by the IUCN Red List [8] as vulnerable. However, protection is needed because illegal hunting remains a threat [71] and, for this reason, there are protected areas for this species as well as captive breeding programmes. An increase in knowledge on crocodile sperm function, including the definition of optimal protocols for sperm analysis, will aid in the development of methods to store semen and use it through assisted reproduction techniques for this and related species. This may allow the establishment of genome resource banks for the conservation of cryopreserved semen samples and their use by means of artificial insemination in genetic management programmes, as is the case with endangered mammals, for which these initiatives have proven very successful [72]. This type of initiatives would contribute to preserve genetic diversity and potentially contribute to assist populations that are currently depleted. In this context, semen analysis and evaluation procedures by CASA-Mot technology described in this study is a significant step towards better understanding of reptile reproduction and towards the conservation of the *C*. *acutus* in captivity and in the wild.

## Conclusions

The American crocodile, *Crocodylus acutus*, is listed as vulnerable due to overexploitation and habitat loss. The reproductive biology of crocodiles has recently attracted attention. Semen analysis is important to assess the reproductive potential of males and CASA technology provides an objective and reliable assessment of the number of motile sperm cells in a sample, as well as for measuring several kinematic variables. Increased knowledge on crocodile sperm function, including the definition of optimal protocols for sperm analysis, will aid in the development of methods to store semen and use it through assisted reproduction techniques for this and related species. In this context, semen analysis and evaluation procedures by CASA-Mot technology described in this study is a initial step towards better understanding of reptile reproduction and towards the conservation of *C*. *acutus* in captivity and in the wild. The new approaches for the analysis of reptile sperm kinematic subpopulations, reflecting quantifiable parameters generated by CASA systems technology, open up possibilities for future assessments of crocodile sperm and will be useful in the future development of assisted reproduction for these species.
